# Inhibition of BTK and SYK attenuates *Porphyromonas gingivalis* -induced activation of the pyroptosis pathway and inflammation in host cells

**DOI:** 10.1080/20002297.2025.2577219

**Published:** 2025-11-06

**Authors:** Alicja Plonczynska, Aureliusz Schuster, Dominika M. Drapala, Tomasz Kaczmarzyk, Magdalena Nowak, Jan Potempa, Aleksander M. Grabiec, Maja Sochalska

**Affiliations:** a Department of Microbiology, Faculty of Biochemistry, Biophysics and Biotechnology, Jagiellonian University, Krakow, Poland; b Doctoral School of Exact and Natural Sciences, Jagiellonian University, Krakow, Poland; c Chair of Oral Surgery, Faculty of Medicine, Jagiellonian University Medical College, Kraków, Poland; d Department of Oral Immunology and Infectious Diseases, University of Louisville School of Dentistry, Louisville, KY, USA

**Keywords:** *Porphyromonas gingivalis*, pyroptosis, periodontitis, kinase, macrophages, fibroblasts

## Abstract

**Background:**

Periodontitisis a chronic inflammatory disease of the oral cavity, primarily driven by periodontopathogens such as *Porphyromonas gingivalis (Pg)*."

**Objective:**

We investigated the therapeutic potential of BTK and SYK inhibitors on the pathological processes induced by two *Pg* strains, ATCC 33277 and W83, in human monocyte-derived macrophages (hMDMs) and human gingival fibroblasts (hGFs).

**Design:**

hMDM and hGF were infected with *Pg* strains and assessed for viability, inflammatory activation, and phenotype, with or without the BTK inhibitor ibrutinib or SYK inhibitor R406, under acute and chronic infection conditions.

**Results:**

Ibrutinib and R406 suppressed *Pg* infection-induced activation of the NLRP3-dependent pyroptosis pathway and IL-1β secretion in hMDMs. Both compounds also significantly reduced IL-6, IL-8, and TNFα release by hMDM in both infection models, regardless of differences between ATCC 33277 and W83 *Pg* strains. Ibrutinib and R406 potently suppressed inflammatory activation of hGFs, including IL-6 and IL-8 production, and NF-κB p65 phosphorylation triggered by the more immunostimulatory ATCC 33277 strain.

**Conclusions:**

The pharmacological inhibition of BTK or SYK mitigates the pyroptotic pathway in hMDMs and exerts a broad anti-inflammatory effect in both hMDMs and hGFs. These results highlight the anti-inflammatory potential of BTK and SYK inhibitors for the treatment of periodontal disease.

## KEY MESSAGES

Ibrutinib and R406 suppress *Pg*-induced pyroptosis signaling and pro-inflammatory cytokine release in hMDMs across different *Pg* strains and infection models.

Ibrutinib uniquely inhibits M1-like polarization and ROS production in hMDMs, while both inhibitors broadly reduce inflammatory activation in hGFs, indicating distinct and complementary immunomodulatory effects.

These findings highlight BTK and SYK inhibition as promising therapeutic strategies to mitigate inflammation and tissue damage in periodontitis (PD), particularly in the context of chronic inflammation, which may be associated with cell senescence.

## Introduction

Periodontitis (PD) is a chronic inflammatory disease of the oral cavity, driven by the presence of periodontopathogens within the gingival tissue. The Gram-negative anaerobic bacterium *Porphyromonas gingivalis* (*Pg*) is frequently implicated in PD pathogenesis [1]. *Pg* utilizes virulence factors such as lipopolysaccharide (LPS), fimbriae, and gingipains to invade host tissues, promote survival, and maintain inflammation that directly causes host tissue destruction. PD progression stems from both persistent bacterial infection and the dysregulation of immune and sentinel cells, notably gingival fibroblasts (GFs) and macrophages [2,3]. GFs, the predominant cells in the gingival tissue, modulate the activity of local immune cells [4]. GFs also produce matrix metalloproteinases (MMPs) and chemokines such as interleukin (IL)-8, thus responding dynamically to the inflammatory environment [3,5]. Macrophages play a central role in the regulation of local inflammatory reactions in the gingiva, particularly a pro-inflammatory M1-like population that secretes IL-6, IL-8, tumor necrosis factor (TNF)-*α*, proteases (e.g., MMPs), reactive oxygen species (ROS), and nitric oxide [6]. In contrast, M2-like macrophages are anti-inflammatory, facilitating tissue repair by secreting IL-4, IL-10 and IL-13 [6]. PD is marked by an imbalance favoring the M1 phenotype, contributing to persistent inflammation and tissue degradation [7–10]. Clinical evidence suggests additional factors contribute to the chronicity of inflammation within the gingiva, particularly pro-inflammatory programmed cell death mechanisms (pyroptosis and necroptosis), which regulate macrophage survival in response to bacterial challenge [11].

Bruton’s tyrosine kinase (BTK) and spleen tyrosine kinase (SYK) are key signaling molecules that regulate immune responses. BTK, acting downstream of Toll-like receptors (TLRs), regulates macrophage polarization and contributes to NF-κB signaling and NLRP3 inflammasome activation [12,13]. BTK inhibitors have been used primarily for the treatment of chronic lymphocytic leukemia, but recent studies have revealed their anti-inflammatory properties in autoimmune and other immune-mediated inflammatory diseases [14]. Ibrutinib, an approved BTK inhibitor, can promote a shift in macrophage polarization from an M1-like to an M2-like phenotype, facilitating tissue repair and the resolution of inflammation [15]. Similarly, SYK, a direct upstream regulator of BTK, modulates TLR signaling pathways [16]. As a key activator of NF-κB signaling, SYK regulates inflammatory cytokine production and also activates the NLRP3 inflammasome [16–18]. R406, a selective SYK inhibitor, has anti-inflammatory activity by reducing IL-6 secretion in synovial fibroblasts [19]. Both inhibitors are emerging as senotherapeutics—agents that selectively target senescent cells or their secretory activity [20,21]. Given the strong association between PD and aging, the application of senotherapeutics in the context of PD has significant clinical potential.

This study aimed to evaluate the therapeutic potential of the BTK inhibitor ibrutinib and the SYK inhibitor R406 in modulating pathological responses induced by *Pg* strains ATCC 33277 and W83 in human monocyte-derived macrophages (hMDMs) and gingival fibroblasts (hGFs).

## Materials and methods

### Cell cultures

We isolated hMDMs from peripheral blood (Regional Blood Donation and Transfusion Center, Krakow, Poland) as previously described [22]. Peripheral blood mononuclear cells (PBMCs) were separated by Pancoll density-gradient centrifugation, and CD14^+^ monocytes were purified using MACS MicroBeads (Miltenyi Biotec). Monocytes were differentiated in RPMI-1640 medium (VWR 392-0427) with 10% fetal bovine serum (FBS; Sigma-Aldrich) and 50 ng/ml GM-CSF (BioLegend) for 7 days. Before experiments, the culture medium was replaced with RPMI-1640 containing 2% FBS without cytokines.

We isolated hGFs from gingival tissue obtained from healthy individuals undergoing the surgical phase of orthodontic treatment at the Department of Periodontology and Clinical Oral Pathology, and the Chair of Oral Surgery, Faculty of Medicine, Jagiellonian University Medical College (Krakow, Poland) as previously described [23]. Tissue collection was approved by the Bioethical Committee of Jagiellonian University (opinion number 1072.6120.74.2023). Healthy donors included in the study (median age [range]: 32 [18–66], male:female ratio 9:8) were non-smokers, without systemic diseases, recent antibiotic therapy, or a history of chemo-/radiotherapy. Cells were cultured in DMEM (Biowest) with 10% FBS (Sigma-Aldrich), 50 U/ml gentamicin (Gibco), and 50 U/ml penicillin/streptomycin (Gibco). For experiments, hGFs (passages 4–9) were cultured in DMEM containing 2% FBS without antibiotics.

### Bacterial strains and infection

Wild-type (WT) *Pg* strains ATCC 33277 and W83 were cultured anaerobically on brain heart infusion (BHI) agar (Becton Dickinson) supplemented with 5 g/l yeast extract (BioShop), 0.5 mg/ml l-cysteine (BioShop), 10 µM hemin (Sigma-Aldrich) and 0.5 μ/ml vitamin K (Sigma-Aldrich) at 37 °C. Before each experiment, cultures grown in BHI broth were adjusted to OD_600_ = 1.0 (~10⁹ CFU/ml). Cells were infected with *Pg* at a multiplicity of infection (MOI) of 20 for 24 h (acute model). For the 7-day model of chronic infection, the cell culture was inoculated again with *Pg* on day 2.

### Inhibitor treatments

Cells were pretreated with ibrutinib or R406 (both from Selleckchem) at 1–10 µM, 30 min before *Pg* infection. For chronic infection, the treatment was repeated on days 2 and 5.

### ELISA and cytotoxicity assay

Supernatants were collected after 24 h or 7 days to quantify lactate dehydrogenase (LDH) activity as a measure of cytotoxicity using the CyQuant LDH Cytotoxicity Assay (Invitrogen) and to quantify IL-1β, TNF-*α*, IL-6, and IL-8 by ELISA (Invitrogen) in technical duplicates. Absorbance was measured using a Flex Station 3 microplate reader (Molecular Devices).

### Flow cytometry

Cell viability was assessed by staining with annexin V-BV421 and propidium iodide (PI). Macrophage polarization was evaluated using antibodies CD80-FITC (Cat# 305206, RRID: AB_314502), CD163-PE/Cyanine 7 (Cat#333614, RRID: AB_2562641) and CD206-BV421 (Cat#321126, RRID: AB_2563839) (all from BioLegend). *Pg* internalization was assessed using bacteria labeled with CellTrace CFSE (Invitrogen). Fluorescence was measured on an LSR Fortessa flow cytometer with FlowJo v10 software.

### Luminescence assays

We assessed cell death kinetics using the RealTime-Glo Annexin V Apoptosis and Necrosis Assay (Promega) up to 72 h post-infection. Caspase-1 activity was tested 3 h post-infection using the Caspase-Glo 1 Inflammasome Assay (Promega), with or without caspase-1 inhibitor YVAD-CHO (Promega), and is presented as net caspase-1 activity (minus inhibitor signal). As the positive control, we used 1 µg/ml *Escherichia coli* LPS plus 20 µM nigericin (both Sigma-Aldrich). We characterized ROS production kinetics using a lucigenin-based assay <2 h post-infection.

### Quantitative PCR (qPCR)

Total RNA was extracted using the ExtractMe RNA Isolation Kit (Blirt) and quantified using a BioPhotometer D30 (Eppendorf). The RNA used as a template for cDNA synthesis with the High-Capacity cDNA Reverse Transcription Kit (Applied Biosystems). Gene expression was quantified by qPCR using SYBR Green Master Mix (Applied Biosystems) on a CFX96 Real-Time PCR system (Bio-Rad). Data were analyzed using the ΔΔCT method with *RPLP0* as the housekeeping gene for normalization. Primers are listed in Appendix Table 1.

### Western blotting

We lysed hMDMs and hGFs directly in Laemmli loading buffer and proteins were separated by SDS-PAGE, transferred to PVDF membranes (Immobilon-PSQ), and blocked with 5% milk (BioShop) in phosphate-buffered saline containing 0.1% Tween-20 (PBST). Blots were probed with primary antibodies against NLRP3 (Cat# 13158, AB_2798134), GSDMD (Cat# 69469, RRID: AB_3697916), caspase-1 (Cat# 3866, AB_2069051), caspase-3 (Cat# 9662, RRID: AB_331439), caspase-4 (Cat# 4450, RRID: AB_1950386), p21 (Cat# 2947, RRID: AB_823586), *p*-p65 NF-κB (Cat# 3033, RRID: AB_331284), and GAPDH (Cat# 2118, RRID: AB_561053), followed by a HRP-conjugated anti-rabbit secondary antibody (Cat# 7074, RRID: AB_2099233) (all antibodies from Cell Signaling Technology). Signals were detected using Pierce ECL substrate (Thermo Fisher Scientific) and Hyperfilm (GE Healthcare), and were imaged using the ChemiDoc MP system (Bio-Rad) with ImageJ software. We used hGFs treated with 1 μM staurosporine (STS; Sigma-Aldrich) as a positive control for caspase-3 activation.

### Statistical analysis

Statistical significance (p ≤ 0.05) was determined by one-way analysis of variance (ANOVA) with Tukey’s *post hoc* test, Kruskal–Wallis with Dunn’s *post hoc* test, or *t*-tests, depending on data distribution (assessed using the Kolmogorov–Smirnov test). Data from at least three independent experiments on MDMs or GFs from different donors (unless stated otherwise) are presented as means ± standard errors (SEM). GraphPad Prism software was used for statistical analysis and data presentation.

### Generative AI tools

Chat Generative Pre-trained Transformer—GPT-4.1 was used for language improvement.

## Results

### P. gingivalis induces pyroptosis signaling in hMDMs

**Figure 1.**
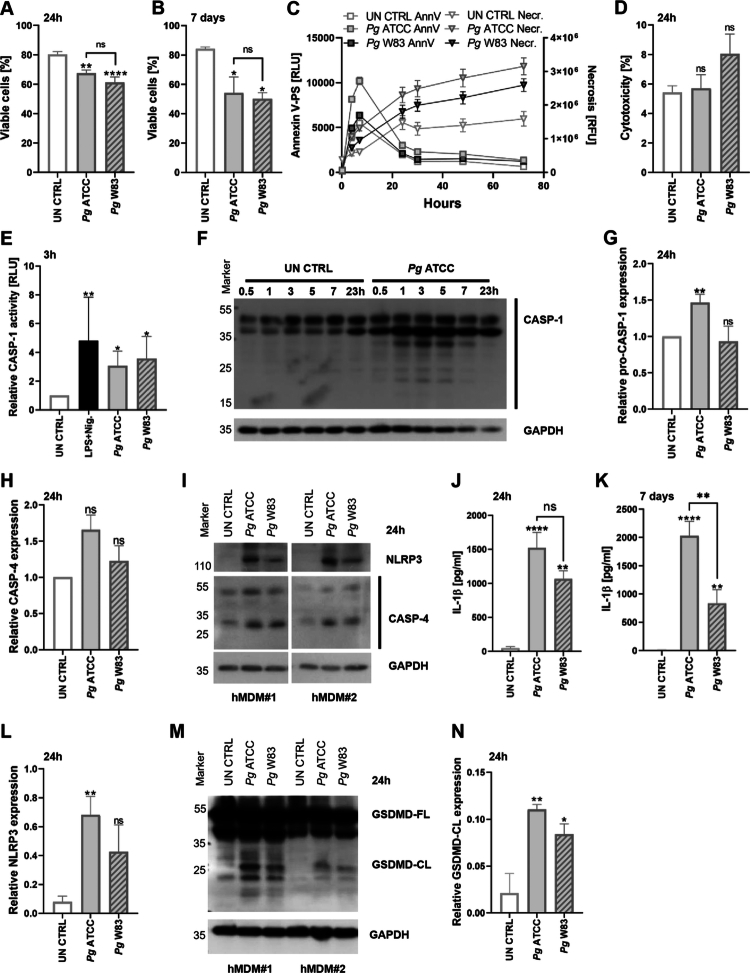
The survival of *Pg*-treated hMDMs decreases due to pyroptosis. (A) Viability of hMDMs infected with *Pg* ATCC 33277 or W83 (MOI = 20), or untreated (UN CTRL), assessed by annexin V/PI staining and flow cytometry after 24 h (*n* = 13) or **(B)** 7 days (*n* = 6). **(C)** Kinetics of phosphatidylserine exposure in hMDMs infected with *Pg*, measured by real-time annexin V binding (RLU) or necrosis staining (RFU) up to 72 h post-infection (*n* = 3). **(D)** Cytotoxicity measured by LDH release from *Pg*-infected hMDMs after 24 h (*n* = 5). **(E)** Caspase-1 activation in hMDMs 3 h after *Pg* infection or stimulation with LPS (*E. coli*) and nigericin, presented as the fold change over untreated control (*n* = 6). **(F)** Representative western blot showing caspase-1 levels. **(G)** Relative expression of pro-caspase-1 (*n* = 6) and **(H)** ~30 kDa active caspase-4 (*n* = 4) in untreated and *Pg*-infected cells, presented as the fold change over untreated control. **(I)** Representative western blot showing NLRP3 and caspase-4 levels in hMDMs from two independent donors. **(J)** IL-1β levels in culture supernatants 24 h (*n* = 11) and **(K)** 7 days (*n* = 12, from *n* = 7 different donors) post-infection, quantified by ELISA. **(L)** Relative expression of NLRP3 (*n* = 3). **(M)** Representative western blot showing levels of the GSDMD full form (GSDMD-FL, ~55 kDa) and cleaved form (GSDMD-CL, ~25 kDa) in hMDMs from two independent donors. **(N)** Relative expression of cleaved GSDMD (GSDMD-CL; *n* = 3). Data are means ± SEM. Statistical significance was determined by one-way ANOVA with Tukey’s *post hoc* test (A), Kruskal–Wallis with Dunn’s *post hoc* test (B, D, E, J, K, L, *N*), or a one-sample *t*-test (G, H) (*p ≤ 0.05, **p ≤ 0.01, ****p ≤ 0.0001; ns = not significant).

To investigate the effect of *Pg* on macrophage viability, primary hMDMs were infected with the commonly used strains ATCC 33277 and W83*,* followed by annexin V/PI staining and the LDH cytotoxicity assay. Both strains increased PI uptake, indicating cell death. The average proportion of viable cells decreased from 80.3% in uninfected controls to 67.6% in cells infected with ATCC 33277 and 61.2% in those infected with W83 after 24 h (Figure 1A). A similar reduction in viability was observed during chronic infection (7 days), with survival dropping from 84.2% in controls to 54.0% and 50.1% in cells infected with ATCC 33277 and W83, respectively (Figure 1B). During the early phase of infection, ATCC 33277 triggered increased phosphatidylserine exposure, detected with annexin V, followed by elevated necrotic staining (Figure 1C). Interestingly, *Pg* infection did not cause significant cytotoxicity, as measured by LDH release after 24 h, suggesting the absence of cell lysis (Figure 1D).

To determine whether *Pg* can trigger the pyroptosis pathway in hMDMs, we examined the expression of caspase-1, caspase-4, NLRP3 and GSDMD, the activity of caspase-1 and caspase-4, and the release of IL-1β. *Pg* infection triggered a slight but significant activation of caspase-1 (Figure 1E, F), accompanied by an increase in total pro-caspase-1 expression in infected cells (Figure 1F, G). The active form of caspase-4, a key component of non-canonical pyroptosis, was more abundant following treatment with ATCC 33277 (Figure 1H, I). Caspase activation was associated with the secretion of IL-1β, observed both 24 h and 7 days after infection (Figure 1J, K). Western blots confirmed NLRP3 inflammasome engagement, with higher levels induced by ATCC 33277 compared to W83 (Figure 1I, L). Additionally, the cleaved, pore-forming form of GSDMD (GSDMD-CL) was detected 24 h after infection with *Pg* (Figure 1M, N). These findings provide strong evidence that the pyroptotic signaling pathway is activated in hMDMs by *Pg* infection, particularly in response to the ATCC 33277 strain.

### BTK and SYK inhibition decrease Pg-driven inflammation in hMDMs

**Figure 2.**
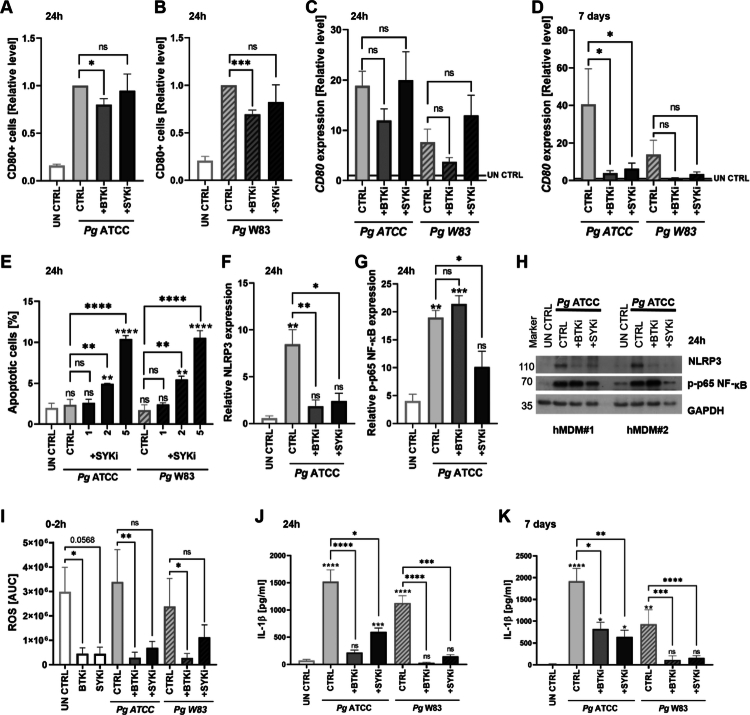
BTKi and SYKi decrease *Pg*-driven inflammation in hMDMs. **(A)** Relative abundance of CD80 on the surface of hMDMs infected with *Pg* ATCC (*n* = 10) or **(B)** *Pg* W83 (*n* = 7) for 24 h following BTKi or SYKi. Data are presented as fold changes over *Pg* control. **(C)** Relative *CD80* mRNA levels after 24 h or **(D)** 7 days of treatment, presented as fold changes over untreated control (UN CTRL). **(E)** Percentage of annexin V^+^ apoptotic hMDMs 24 h after co-treatment with *Pg* and SYKi (*n* = 3). **(F)** Relative expression of NLRP3 (*n* = 3) and **(G)** phosphorylated p65 NF-κB (*p*-p65; *n* = 3). **(H)** Representative western blots showing levels of NLRP3 and *p*-p65 NF-κB in hMDMs from two independent donors. **(I)** Area under the curve (AUC) analysis of ROS production in untreated control (UN CTRL) or *Pg-*infected hMDMs, with or without BTKi or SYKi, within the first 2 h post-infection. **(J)** IL-1β levels in supernatants 24 h (*n* = 14–18, from *n* = 8 different donors) or **(K)** 7 days (*n* = 4–6, from *n* = 3 different donors) after infection, quantified by ELISA. BTK and SYK inhibitors were provided at a concentration of 5 μM unless otherwise stated. Data are means ± SEM. Statistical significance was determined by one-way ANOVA with Tukey’s *post hoc* test (C-G), Kruskal–Wallis with Dunn’s *post hoc* test (I-J), or a one-sample *t*-test (A, B) (*p ≤ 0.05, **p ≤ 0.01, ***p ≤ 0.001, ****p ≤ 0.0001; ns = not significant).

To investigate the effect of inhibiting BTK and SYK on macrophage survival and signaling, we treated hMDMs with ibrutinib, which specifically inhibits BTK (BTKi), or R406, which specifically inhibits SYK (SYKi). Consistent with its mode of action, ibrutinib rapidly reduced the proportion of CD80^+^ cells, a marker of the M1-like phenotype, at both the surface protein and mRNA levels (Figure 2A–D), with strong, significant reduction continuing until 7 days after treatment (Figure 2D). In contrast, R406 affected *CD80* expression only in the chronic infection model (Figure 2D). Interestingly, both inhibitors also reduced the population of CD163^+^CD206^+^ M2 macrophages, measured at the surface level 24-h post-infection (Appendix Figure 1). R406 also promoted apoptosis in infected cells, suggesting a redirection from inflammatory pyroptosis to non-inflammatory programmed cell death (Figure 2E), whereas ibrutinib slightly reduced cell death (Appendix Figure 2).

Both inhibitors reduced ATCC 33277-induced NLRP3 expression. However, only R406 suppressed phosphorylation of the p65 subunit of NF-κB, whereas ibrutinib had no significant effect on this pathway (Figure 2F–H). Both inhibitors decreased constitutive ROS production but only ibrutinib reduced ROS generation to the baseline in *Pg*-infected cells (Figure 2I). *Pg* infection itself did not strongly induce ROS generation (Figure 2I).

**Figure 3.**
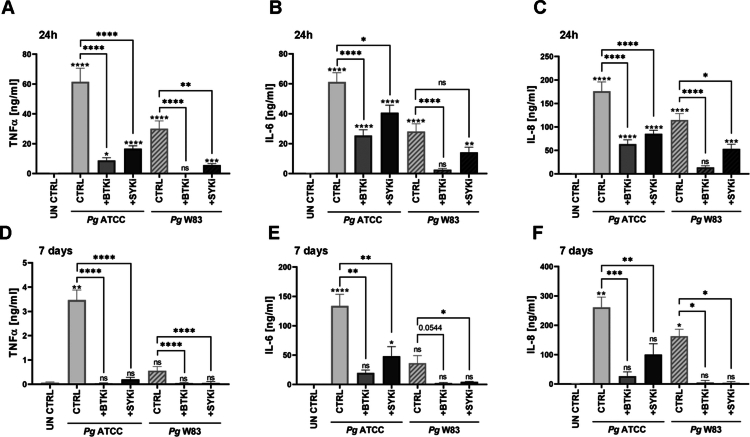
BTKi and SYKi decrease *Pg*-induced secretion of pro-inflammatory cytokines in hMDMs. **(A)** TNFα (*n* = 17–21, from *n* = 9 different donors), **(B)** IL-6 (*n* = 16–20, from *n* = 9 different donors) and **(C)** IL-8 (*n* = 16–20, from *n* = 9 different donors) levels in supernatants 24 h after BTKi or SYKi and infection with *Pg* ATCC or W83, quantified by ELISA. **(D)** TNFα (*n* = 5, from *n* = 3 different donors), **(E)** IL-6 (*n* = 4–6, from *n* = 3 different donors) and **(F)** IL-8 (*n* = 4–6, from *n* = 3 different donors) levels in supernatants 7 days after infection, quantified by ELISA. BTK and SYK inhibitors were provided at a concentration of 5 μM. Data are means ± SEM. Statistical significance was determined by Kruskal–Wallis with Dunn’s *post hoc* test (*p ≤ 0.05, **p ≤ 0.01, ***p ≤ 0.001, ****p ≤ 0.0001; ns = not significant).

The anti-pyroptotic and anti-inflammatory effects of BTKi and SYKi were further supported by cytokine profiling. Ibrutinib strongly inhibited IL-1β secretion 24 h (Figure 2J) and 7 days post-infection (Figure 2K). IL-1β release was reduced by both *Pg* strains and at both time points following treatment with either inhibitor. Additionally, both inhibitors suppressed the secretion of pro-inflammatory cytokines TNFα and IL-6, as well as chemokine IL-8 (Figure 3A–C). This inhibitory effect persisted throughout the prolonged infection (Figure 3D–E).

Taken together, our findings demonstrate that pharmacological BTKi and SYKi limit NLRP3 inflammasome activation and reduce the pro-inflammatory response of hMDMs, promoting the resolution of inflammation.

### Pg infection does not alter GF survival but induces an inflammatory response

**Figure 4.**
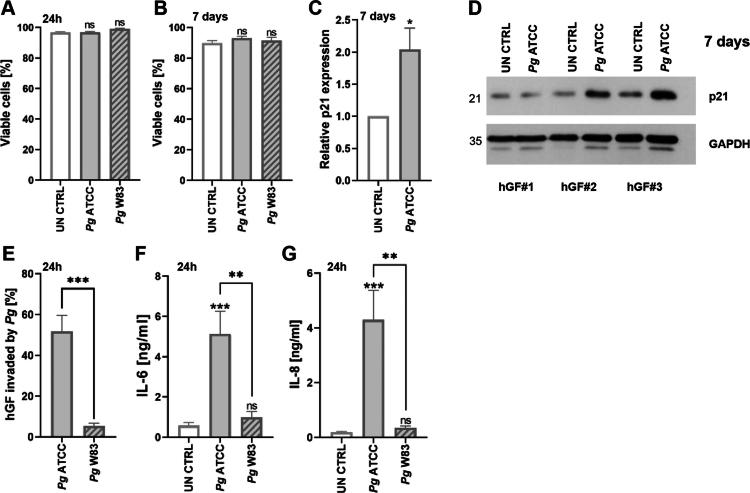
*Pg* infection does not alter hGF survival but induces an inflammatory response. **(A)** Viability of hGFs infected with *Pg* ATCC 33277 or W83, or left untreated (UN CTRL), assessed by annexin V/PI staining after 24 h (*n* = 3–8) or **(B)** 7 days (*n* = 6–10). **(C)** Relative expression of p21 in untreated and *Pg* ATCC 33277-infected cells 7 days after infection, presented as fold change over untreated control (*n* = 7). **(D)** Representative western blots showing p21 levels in untreated and *Pg* ATCC 33277-infected cells 7 days after infection in hGFs from three independent donors. **(E)** Intracellular uptake of CFSE-labeled *Pg* ATCC 33277 and W83 in hGFs 24 h post-infection (*n* = 6). **(F)** IL-6 and **(G)** IL-8 levels in supernatants 24 h post-infection (*n* = 6), quantified by ELISA. Data are means ± SEM. Statistical significance was determined by one-way ANOVA with Tukey’s *post hoc* test (A, B, F, G), an unpaired *t*-test (E), or a one-sample *t*-test (C) (*p ≤ 0.05, **p ≤ 0.01, ****p ≤ 0.0001; ns = not significant).

*Pg* did not significantly affect the survival of GFs in either the acute or chronic infection models (Figure 4A, B). However, prolonged exposure to ATCC 33277 increased expression of the cell cycle regulator p21, indicating potential cell cycle arrest (Figure 4C, D). Notably, there was a marked difference in bacterial internalization between the two strains. ATCC 33277 invaded GFs at levels comparable to those seen in macrophages (Appendix Figure 3), whereas W83 showed minimal internalization (Figure 3E). Likewise, the secretion of pro-inflammatory IL-6 and IL-8 was strongly induced by ATCC 33277 but only weakly by W83 (Figure 4F, G). Taken together, these findings suggest that W83 evades detection by sentinel cells while selectively eliciting a response from specialized immune cells such as macrophages. This may enable W83 to persist within the gingival tissue for extended periods, thereby promoting its survival and proliferation.

### BTKi and SYKi decrease Pg-driven inflammation in hGF

**Figure 5.**
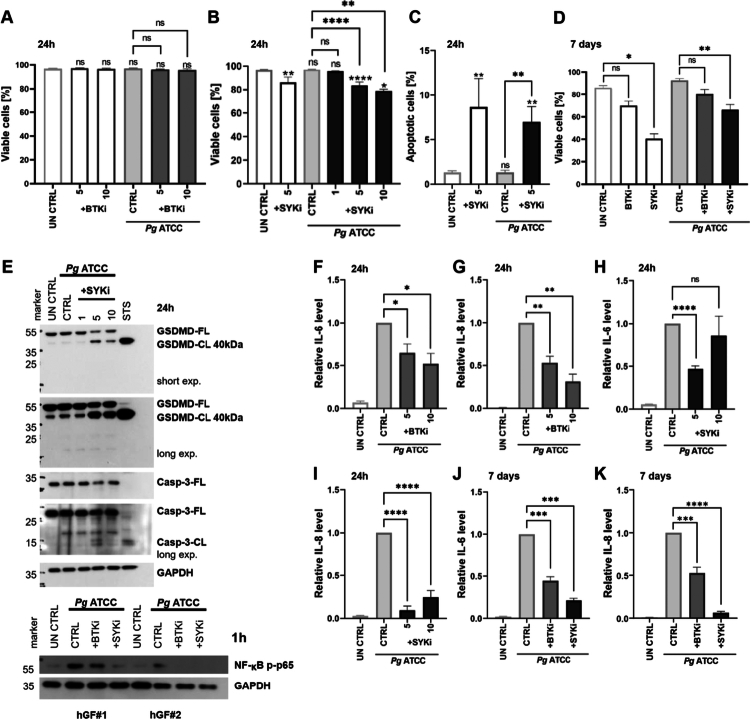
BTKi and SYKi reduce *Pg*-induced inflammation in hGFs. **(A)** Viability of hGFs co-treated with *Pg* ATCC 33277 and BTKi (*n* = 7) or **(B)** *Pg* ATCC 33277 and SYKi (*n* = 3–15) assessed by annexin V/PI staining 24 h post-infection. **(C)** Percentage of annexin V^+^ apoptotic hGFs 24 h after co-treatment with *Pg* and SYKi (*n* = 8–17). **(D)** Viability of hGFs co-treated with *Pg* ATCC 33277 and BTKi or SYKi after 7 days, assessed by annexin V/PI staining (*n* = 4). **(E)** Representative western blots showing (upper panels) caspase-3 and GSDMD levels 24 h after co-treatment with SYKi and *Pg* ATCC 33277, with 1 μM staurosporine (STS) as a positive control, and (lower panels) *p*-p65 NF-κB levels after 1 h. **(F)** Relative IL-6 (*n* = 4) and **(G)** IL-8 (*n* = 4) levels in supernatants 24 h after infection with *Pg* ATCC 33277 and BTKi, quantified by ELISA, presented as fold change over *Pg* control. **(H)** Relative IL-6 (*n* = 7–16) and **(I)** IL-8 (*n* = 8) levels in supernatants 24 h after infection with *Pg* ATCC 33277 and SYKi, quantified by ELISA, presented as fold change over *Pg* control. **(J)** Relative IL-6 (*n* = 6) and **(K)** IL-8 (*n* = 6) levels in supernatants 7 days after infection with *Pg* ATCC 33277 and BTKi or SYKi, quantified by ELISA, and presented as fold change over *Pg* control. BTK and SYK inhibitors were provided at a concentration of 5 μM unless otherwise stated. Data are means ± SEM. Statistical significance was determined Kruskal–Wallis with Dunn’s *post hoc* test (A-D) or a one-sample *t*-test (F-K) (*p ≤ 0.05, **p ≤ 0.01, ***p ≤ 0.001, ****p ≤ 0.0001; ns = not significant).

We treated hGFs with ibrutinib and R406 to determine whether and how BTKi and SYKi affect *Pg*-induced fibroblast activation. BTKi did not affect hGF viability (Figure 5A) whereas SYKi slightly reduced hGF viability in the acute model and caused a significant decrease in survival during chronic infection (Figure 5B, D). SYKi-induced apoptosis was confirmed by annexin V/PI staining (Figure 5C), and the 7-day treatment exacerbated cell death, while BTKi only slightly reduced the number of viable cells (Figure 5D).

Western blots provided evidence of SYKi-induced apoptosis by confirming the cleavage of caspase-3 into its active p12/p17 subunits and the cleavage of GSDMD into a 40-kDa inactive fragment (Figure 5E). Both BTKi and SYKi inhibited the pro-inflammatory activation of hGFs induced by ATCC 33277. BTKi also reduced the secretion of IL-6 and IL-8 by ~50%, and SYKi almost completely abolished IL-8 production and markedly decreased IL-6 levels (Figure 5F–I). These immunomodulatory effects were observed in both the acute and chronic infection models (Figure 5J, K).

## Discussion

*Pg* is a keystone pathogen in the disruption of oral cavity homeostasis during PD. We investigated the effects of two common *Pg* strains on hMDMs and hGFs, focusing on inflammation and cell death. Strains ATCC 33277 and W83 are widely used in PD research and represent the diversity of clinical isolates. ATCC 33277-like strains are found in both healthy individuals and PD patients, whereas W83-like strains are more frequently associated with disease [24]. ATCC 33277, characterized by long fimbriae and the absence of a capsule, is considered less virulent [24–26], whereas W83 is both encapsulated and afimbriate [27–29]. These differences affect host–pathogen interactions and may cause inconsistencies across PD models. The notably stronger immune response triggered by ATCC 33277 compared to W83 in both hMDMs and hGFs, as demonstrated in this study, underscores the significant biological differences between these strains and suggests the greater ability of W83 to evade the host immune response.

Previous studies have shown that pro-inflammatory forms of programmed cell death (pyroptosis and necroptosis) can be triggered by *Pg* and its virulence factors [11]. Here, we compared the effects of *Pg* on the viability of hMDMs and hGFs, two major cellular regulators of inflammation in PD. Both *Pg* strains significantly increased the uptake of PI by hMDM, indicating a loss of membrane integrity and activation of the pyroptotic pathway involving NLRP3, caspase-1, caspase-4, GSDMD, and the secretion of IL-1β. However, LDH release did not increase after infection with either strain. Although LDH release is considered a hallmark of pyroptosis, emerging evidence suggests that pyroptotic signaling can occur without complete cell lysis [30]. For this reason, some researchers suggest distinguishing the activation of the pyroptosis/inflammasome pathway, which results in cell hyperactivation from pyroptosis itself, understood as a form of cell death [31,32]. This sub-lethal pyroptosis pathway activation has been observed in cells where GSDMD pore formation enables cytokine release without irreversible membrane rupture [33,34]. In another study, Gaidt et al. [35] demonstrated that human monocytes can activate the alternative inflammasome pathway in response to TLR4 stimulation by LPS, without inducing cell death. However, the expression of pyroptosis-associated markers, such as caspase-1 activation and IL-1β secretion, was weaker compared to the classical two-step pyroptosis pathway. Similarly, Wolf et al. [36] observed that in bone marrow-derived macrophages (BMDMs), exposure to bacterial peptidoglycan fragments led to low-level but sustained NLRP3 activation and IL-1β secretion, also without triggering cell death. Consistent with these findings, we found that *Pg*-infected hMDMs activated pro-inflammatory pathways, including pyroptosis, but with minimal cytoplasmic leakage (associated with cell death). It is unclear whether this reflects a small population of cells undergoing full pyroptosis or reversible, spatially restricted GSDMD pore formation in a larger proportion of the cells in the hyperactivation state.

The viability of hGFs was unaffected by acute (24 h) or chronic (7 day) *Pg* infection. Previous studies suggest hGF numbers may decline in PD patients due to apoptosis, but this effect was primarily observed at much higher bacterial doses (MOI = 100–900) than used in our study (MOI = 20), which better reflects physiological conditions [37]. *Pg* LPS combined with ATP can trigger hGFs to release classical pyroptosis markers such as caspase-1 and IL-1β [38]. But despite the robust inflammatory response observed in our study, evidenced by elevated IL-6 and IL-8 levels, we did not detect the secretion of IL-1β (data not shown). The depletion of hGFs in PD, as observed in other studies, may also reflect impaired regenerative capacity due to inflammation-induced cellular senescence. We found that a 7-day exposure to *Pg* was sufficient to transiently inhibit cell cycle progression, as evidenced by increased p21 levels. This aligns with clinical findings showing senescent fibroblasts in gingival biopsies from PD patients [39]. Moreover, pyroptotic macrophages in inflamed gingival tissue can promote senescence in neighboring gingival fibroblasts, indicating that inflammatory cross-talk contributes to tissue degeneration in PD [40].

BTK regulates inflammatory processes in gingival macrophages. Previous studies have demonstrated elevated BTK expression in refractory periapical periodontitis, correlating with bone resorption and PD progression [41]. BTK mediates downstream signaling from RANKL and bacterial components such as LPS, influencing macrophage polarization and pyroptosis via NLRP3 activation [12,42]. In this study, the pharmacological inhibition of BTK with ibrutinib reduced the pro-inflammatory activity of hMDMs significantly by limiting M1-like polarization and NLRP3 signaling. Ibrutinib also limited pro-inflammatory activation of hGFs, without compromising cell survival. Given the central role of BTK in inflammation and bone remodeling, BTKi has potential for PD therapy by mitigating host-mediated tissue damage via multiple *Pg*-modulated pathways.

SYK is another intracellular kinase involved in macrophage inflammatory responses and is activated by bacterial components such as LPS. SYK promotes NF-κB signaling via p65 and p50 subunits, thus inducing the expression of pro-inflammatory genes [18]. We demonstrated that the SYK inhibitor R406 suppressed NF-κB activation and inflammatory cytokine release in both hMDMs and hGFs, and inhibited the expression of NLRP3. The mechanism of SYK-mediated NLRP3 activation relies on the phosphorylation of ASC, part of the inflammasome complex [17]. SYK is necessary for macrophage phagocytic activity, and works by upregulating ROS-generating enzymes [43]. In our *Pg* infection model, R406 modestly reduced ROS levels but did not impair bacterial uptake by hMDMs or hGFs (Appendix Figure 4). In mouse models of ligature-induced periodontitis, myeloid-specific *Syk* knockout reduced bone loss without affecting pro-inflammatory cytokines or osteoclast numbers [44]. Other SYK inhibitors, like GS-9973, block osteoclast differentiation and mineral resorption, underscoring SYK’s central roles in PD-related bone destruction [44].

SYK functions upstream or parallel to BTK in regulating inflammation and inflammasome activation, suggesting that inhibiting these kinases results in redundancy [45]. However, evidence indicates that, although ibrutinib and R406 target overlapping immune pathways, their inhibitory effects can differ. One study highlights the difference in inflammasome pathway regulation: while BTK is essential for NLRP3 activation, SYK deficiency only partially impairs its activation [45]. In another study comparing ibrutinib and R406, it was demonstrated that R406 has a stronger pro-apoptotic effect through Mcl-1 downregulation than ibrutinib in chronic lymphocytic leukemia (CLL) samples [46]. This difference is also evident in the senescence field, where R406, due to its pro-apoptotic functions, is identified as senolytic, whereas ibrutinib, with more limited pro-apoptotic activity, acts as a senomorphic or senoblocker [20,47]. Similar differences in the actions of both inhibitors were observed in our study, especially in the hGF, where R406 caused decreased cell survival due to apoptosis.

## Conclusions

Ibrutinib and R406 significantly limited the inflammatory responses of hMDMs and hGFs by suppressing NLRP3 activation in hMDMs and cytokine secretion in both cell populations. Our findings support BTK and SYK inhibition as promising therapeutic strategies for reducing inflammation and tissue damage in PD, particularly in light of growing evidence that cellular senescence drives chronic inflammation and tissue degeneration in PD.

## Supplementary Material

Supplementary material Appendix Table 1. Sequences of primers used for qPCR.
