# Supplementary material for: Inhibition of BTK and SYK attenuates Porphyromonas gingivalis -induced activation of the pyroptosis pathway and inflammation in host cells
Source: J Oral Microbiol. 2025 Nov 6;17(1):2577219. doi: 10.1080/20002297.2025.2577219 (PMC12599364; doi:10.1080/20002297.2025.2577219)
Supplement: Supplementary material — Appendix Table 1. Sequences of primers used for qPCR. [file ZJOM_A_2577219_SM7875.docx]

**Appendix Materials**

**Appendix Table 1. Sequences of primers used for qPCR.**

| **Gene** | **Forward primer** | **Reverse primer** |
| --- | --- | --- |
| ***CD80*** | CTGCCTGACCTACTGCTTTG | GGCGTACACTTTCCCTTCTC |
| ***CD206*** | GCAGAAGGAGTAACCCACCC | TGGCAAATGAAGGCGTTTGG |
| ***RPLP0*** | GCGTCCTCGTGGAAGTGACATCG | TCAGGGATTGCCACGCAGGG |

**B**

**A**

**
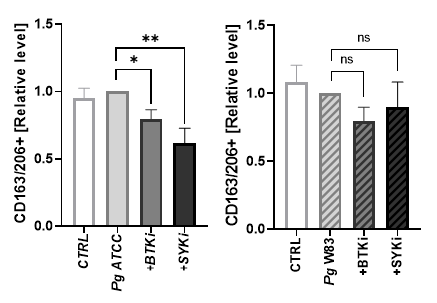
**

**Appendix Figure 1. Macrophage polarization upon BTKi or SYKi treatment.** Relative abundance of CD163^+^CD206^+^ on the surface of hMDMs infected with **(A)** *Pg* ATCC (n = 9) or **(B)** *Pg* W83 (n = 6) following BTKi (5 μM ibrutinib) or SYKi (5 μM R406) for 24 h. Flow cytometry data presented as fold change over *Pg*-infected control. Data are means ± SEM. Statistical significance was determined using a one-sample *t*-test (*p ≤ 0.05, **p ≤ 0.01; ns = not significant).

**Appendix Figure 2. Macrophage viability upon BTKi treatment.** Viability of hMDMs co-treated with *Pg* ATCC 33277 or W83 and BTKi (5 μM ibrutinib), assessed by annexin V/PI staining after 24 h (n = 9–12). Data are means ± SEM. Statistical significance was determined by one-way ANOVA with Tukey’s *post hoc* test (*p ≤ 0.05, **p ≤ 0.01; ns = not significant).

**
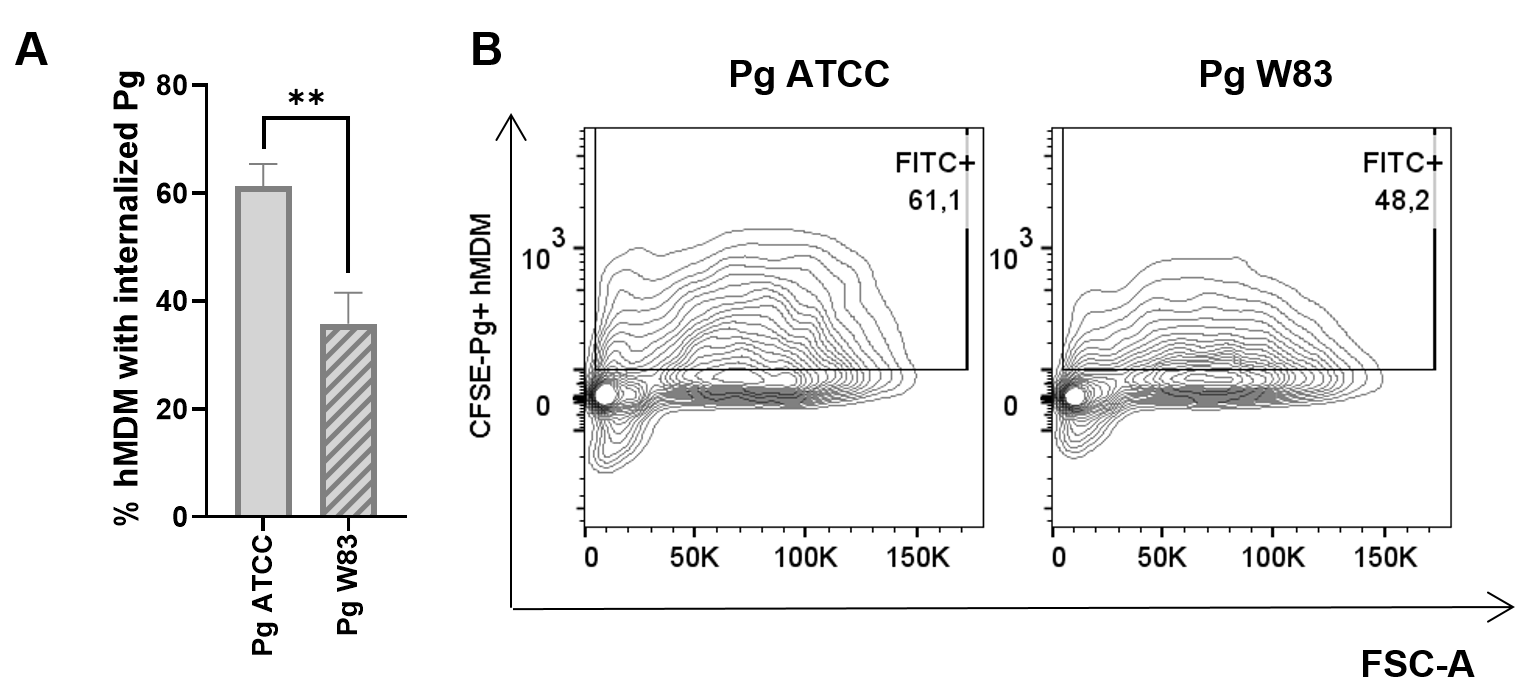
**

**Appendix Figure 3. The internalization of *Pg* by macrophages.** Intracellular uptake of CFSE-labeled *Pg* ATCC 33277 and W83 by hMDMs 24 h post-infection (n = 10). **(A)** Data presented as histograms (means ± SEM). Statistical significance was determined using a *t*-test (**p ≤ 0.01; ns = not significant). **(B)** Data presented as scatter plots.

**C**

**B**

**A**

**
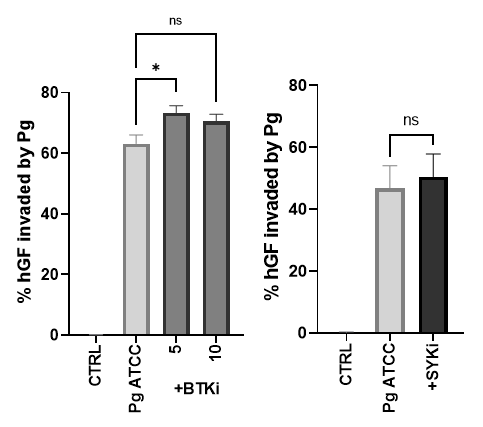
**

**Appendix Figure 4. The internalization of *Pg* by hMDMs and hGFs upon BTKi or SYKi treatment. (A)** Intracellular uptake of CFSE-labeled *Pg* ATCC 33277 and W83 by hMDMs co-treated with BTKi (5 μM ibrutinib) or SYKi (5 μM R406) measured by flow cytometry 24 h post-infection (n = 4–8). **(B)** Intracellular uptake of CFSE-labeled *Pg* ATCC 33277 by hGFs co-treated with BTKi (n = 5) or **(C)** SYKi (n = 4–6) 24 h post-infection. Data are means ± SEM. Statistical significance was determined by Kruskal–Wallis with Dunn’s *post hoc* test, one-way ANOVA with Tukey’s *post hoc* test or a *t*‑test (*p ≤ 0.05; ns = not significant).


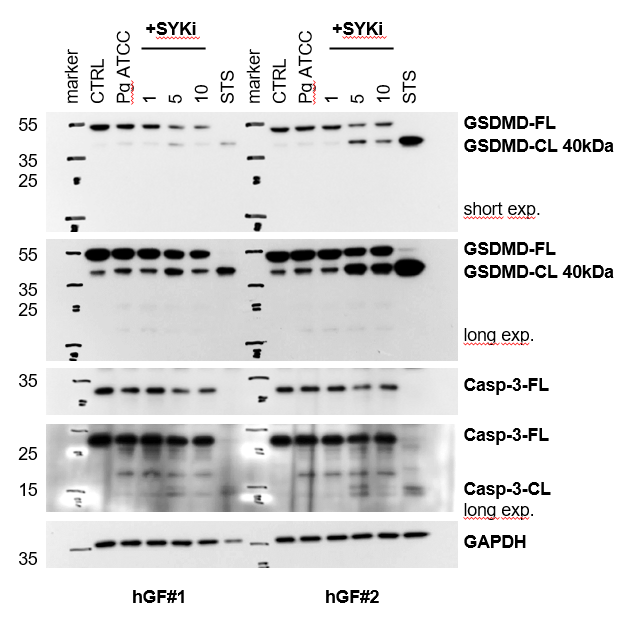

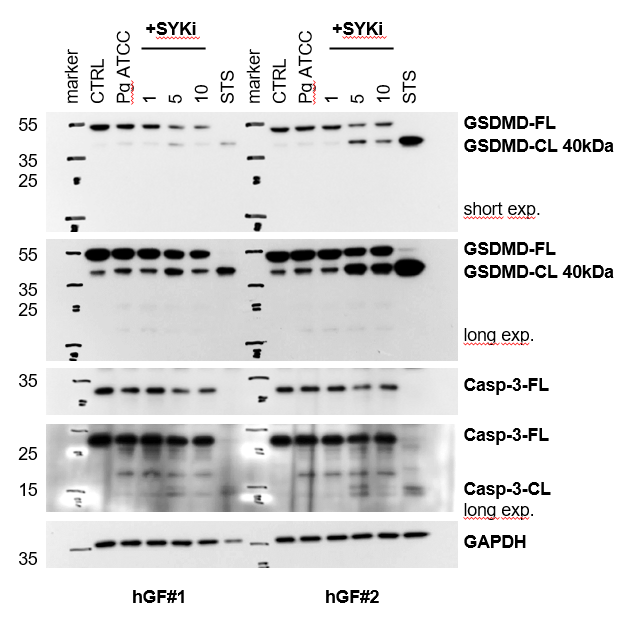


**Appendix Figure 5. Caspase-3 and GSDMD expression in hGFs.** Representative western blots showing levels of caspase-3 and GSDMD 24 h after co-treatment with *Pg* ATCC 33277 and SYKi (5 μM R406), with 1 μM staurosporine (STS) as a positive control.
